# Interactions of BRCA1-mutated Breast Cancer Cell Lines with Adipose-derived Stromal Cells (ADSCs)

**DOI:** 10.1007/s10911-021-09493-4

**Published:** 2021-07-06

**Authors:** Adelina Plangger, Werner Haslik, Barbara Rath, Christoph Neumayer, Gerhard Hamilton

**Affiliations:** 1 Department of Vascular Surgery, Medical University of Vienna, Vienna, Austria; 2 Department for General Gynecology and Gynecologic Oncology, Medical University of Vienna, Vienna, Austria

**Keywords:** Breast cancer, BRCA1 mutation, Lipofilling, Adipose-derived stromal cells, Proliferation, Migration

## Abstract

Lipofilling may constitute a technique to assist reconstruction of breasts following prophylactic mastectomy for patients with mutated BRCA1 or BRCA2 genes. However, to date it is not clear whether adipose-derived stromal cells (ADSCs) increase the risk of tumor initiation and progression in this situation. Therefore, the interactions of BRCA1 mutated breast cancer cell lines with normal ADSCs were investigated in the present study. Characteristics of MDA-MB-436 (BRCA1 c.5277 + 1G > A) and HCC1937 (BRCA1 p.Gln1756.Profs*74) were compared to MDA-MB-231 and T47D BRCA1/2 wild-type breast cancer cell lines. ADSCs were cultivated from lipoaspirates of a panel of BRCA1/2- wildtype patients. Interactions of conditioned medium (CM) of these cells with the breast cancer lines were studied using proliferation and migration assays as well as adipokine expression western blot arrays. CM of ADSCs exhibit a dose-dependent stimulation of the proliferation of the breast cancer cell lines. However, of the ADSC preparations tested, only 1 out of 18 samples showed a significant higher stimulation of BRCA1-mutated MDA-MB-436 versus wildtype MDA-MB-231 cells, and all CM revealed lower stimulatory activity for BRCA1-mutated HCC1937 versus wildtype T47D cells. Additionally, migration of breast cancer cells in response to CM of ADSCs proved to be equivalent or slower for BRCA1/2 mutated versus nonmutated cancer cells and, with exception of angiopoietin-like 2, induced expression of adipokines showed no major difference. Effects of media conditioned by normal ADSCs showed largely comparable effects on BRCA1-mutated and wildtype breast cancer cell lines thus advocating lipofilling, preferentially employing allogeneic non-mutated ADSCs.

## Introduction

The most common cancer among women is breast cancer (BC) and around 5–10% of BC cases are hereditary. A quarter of all these cases are linked to germline mutations such as breast cancer genes (BRCA) 1 and 2 which function as tumor suppressor genes [1]. Mutations in these genes are strongly related with breast and ovarian cancer, but also pancreas or prostate cancer [2]. In Ashkenazi Jews the prevalence of these mutations is higher compared to the general population (1/40 individuals versus 1/300 individuals) [3]. Patients with mutated breast cancer genes (BRCA) 1 or 2 have a lifetime risk of developing BC up to 72% and 69%, respectively. BRCA-associated cancer patients often show a more aggressive form of the disease compared to sporadic cancer cases. Additionally, breast cancer with BRCA 1 is more often of high grade and triple negative resulting in a poorer overall survival [1]. Patients with BRCA2 mutations are often estrogen-receptor positive and show an increased risk compared to other cancer types.

Bilateral prophylactic mastectomy reduces the risk of BC by more than 90% in patients bearing BRCA1/2 mutations. In the US over 100.000 women undergo this form of operation each year. Within 4 years the rate of bilateral prophylactic mastectomy increased by approximately 35.7% only in the US. This operation is recommended for women between the age of 25–30 years [4]. After mastectomy, a variety of reconstructive techniques can be used to reduce the surgical burden of the patients and to provide a higher quality of life. These strategies include implants, autologous tissues or the combination of both. Lipofilling has been demonstrated as an effective breast reconstruction technique which can be combined with implants and flaps [5]. This method has several benefits as fat is an autologous tissue, it is soft and malleable and, furthermore, available in sufficient quantity in the body [6]. The benefit of lipofilling is the usage of the patients own fatty tissue that contains numerous adipose-derived stromal cells (ADSCs). Basically, these mesenchymal stromal cells have the potential to differentiate into adipocytes and other mesodermal tissue types and replace them. Although this type of stromal cells is often confused with stem cells it does not show the same properties as embryonal pluripotent stem cells [7].

ADSC have the ability of multilineage differentiation into bone, cartilage, several muscle types, blood vessels, nerves as well as skin. They are also associated with adipogenesis in the transplanted tissue and revascularization via paracrine effects. These cells can be easily harvested by standard liposuction without the need for further cultivation [8]. The fat is harvested by liposuction from a suitable donor site of the patient (such as thighs or abdomen) and is then centrifuged in order to remove blood and to enrich the preparation with adipocytes. The ADSC-enriched fat is then injected into the breast for reshaping purposes [9]. Due to a slower metabolic activity rate, progenitor cells survive longer without nutrition and consume less oxygen compared to mature adipocytes. Additionally, they are more resistant to hypoxic and traumatic damage due to the processing of the harvested fat as mature adipocytes are more fragile and are not as persistent [7, 8]. However, the side effects of injection of fat are necrosis, formation of cysts and hardening of tissue which could be mistaken as cancerous calcifications. However, the reabsorption rate of the injected fat tissue varies for every patient and may be as high as in 30% of the cases [9].

Currently, little is known about the interaction between ADSCs and BRCA-mutated normal and cancer cells. It is assumed that growth factors and cytokines secreted by ADSCs have a crucial impact on cancer initiation, progression and metastasis [5]. Several studies raised the concern of a potential contribution of the ADSCs-conditioned microenvironment to cancer development and/or as a possible additional stimulation of tumor growth [10]. However, clinical studies and experimental data failed to provide evidence of an increased risk of tumor neoformation or recurrence in BC patients [7]. Contrary to nonhereditary BC, lipofilling in BRCA1/2 patients employing ADSCs may pose additional risks which has been not fully investigated so far. A study by Zhao et al. reported a tumor-promoting effect of ADSCs in which BRCA1 was inactivated using CRISPR/Cas9 knockdown [11]. These cells effect a approximately twofold growth stimulation of breast cancer cell lines and an increase in the inflammatory mediator IL-8 thus provoking a more malignant tumor phenotype.

In the present study we compared the BRCA1/2 wildtype BC cell lines MDA-MB-231 and T47D with two BRCA1-mutated cell lines, namely MDA-MB-436 and HCC1937. Cell lines were tested for their alterations of proliferation and migration as well as for changes in the expression of selected adipokines in response to CM derived from a panel of ADSCs. For this investigation we used normal ADSCs due to the nonavailability of BRCA1-mutated ADSCs, questionable validity of the phenotype of genetically modified ADSCs and the putative use of allogeneic normal ADSCs that exhibit low immunogenicity.

## Patients and Methods

### Isolation, Characterization and Differentiation of ADSCs 

ADSCs were recovered from BRCA1/2 wildtype female patients following liposuction with the written consent of the patients according to the Ethics Approval 366/2003 of the Ethics Committee of the Medical University of Vienna, Vienna, Austria. Fat particles resulting from aspiration through the 12 gauge cannulas were incubated for 5 days in RPMI-1640 medium (Seromed, Berlin, Germany) supplemented with 30% fetal bovine serum (Seromed) and antibiotics (Sigma-Aldrich, St. Louis, MO, USA), thereafter fat tissue was discarded and the ADSCs that became attached to the tissue culture flasks were further cultivated and expanded using 10% FBS. The ADSCs were characterized by flow cytometry by testing the expression of CD73, CD90, and CD105 and negative reactivity for CD34 (all antibodies from Biolegend, San Diego, CA, USA) using a Cytomics FC500 FACS (Beckman Coulter Germany GmbH, Krefeld, Germany) as described previously [7]. Antibodies and isotype controls were from Biolegend (San Diego, CA, USA) and secondary reagents from Sigma-Aldrich). Data analysis and histogram overlays were done employing the Kaluza flow analysis software (Beckman Coulter). CM of the ADSCs were prepared by harvesting supernantants of ADSCs from confluent cells which were kept for a duration of three days.

### BRCA1-mutated Breast Cancer Cell Lines and Normal Controls

 The BRCA1-mutated breast cancer cell lines MDA-MB-436 and MDA-MB-231 as well as the control lines T47D and HCC1937 were cultured in RPMI-1640 medium supplemented with 10% fetal bovine serum (FBS, Seromed, Berlin, Germany) and on confluence cells were detached with trypsin/EDTA (Sigma-Aldrich) and cell numbers counted with a LUNA cell counter (Biozym, Vienna, Austria).

### **Adipokine Western Blot Arrays**

Adipokine markers were analyzed using the ARY024 Proteome Profiler Array (R&D Systems, Minneapolis, MN, USA) according to manufacturer’s instructions. Experiments were done in duplicate and individual membranes calibrated using the included protein controls. Arrays were evaluated using ImageJ and Origin 9.1 software (OriginLab, Northampton, MA, USA).

### Cell Proliferation Assays

 1 × 10^4^ cells in 100 µl medium were distributed to wells of 96-well microtiter plates (TPP Techno Plastic Products, Trasadingen Switzerland) and ten twofold dilutions of CM were added in triplicate starting with a 1:1 ratio of CM to culture medium. Assays were at least performed in triplicate. The plates were incubated for four days and viable cells detected using a modified 3-(4,5-Dimethylthiazol-2-yl)-2,5-diphenyltetrazoliumbromid (MTT) assay (EZ4U, Biomedica, Vienna, Austria). Test results were calculated using Origin 9.1 software (OriginLab, Northampton, MA, USA).

### **Migration Assay**

Cell lines MDA-MB-436, MDA-MB-231 and HCC1937 were kept in 6-well plates (TPP) in 3 ml medium until confluency was reached. T47D showed no migratory capacity under these conditions and was not considered for these experiments. Then, 2 perpendicular scratches were set to remove cells using a plastic tip and wells were supplemented with 1 ml of control medium or respective ADSC-CMs and further incubated under tissue culture conditions. Pictures were taken using a light microscope (magnification 40x) for 3 successive days and areas not covered by cells calculated by ImageJ software (imagej.net) for several positions. Migratory capacity is presented as area newly covered by the breast cancer cells.

### **Statistical Analysis**

Statistical significance was determined by t-tests and *P* < 0.05 regarded as significant difference.

## Results

### ADSC-dependent Growth Stimulation of the Breast Cancer Cell Lines

**Fig. 1:**
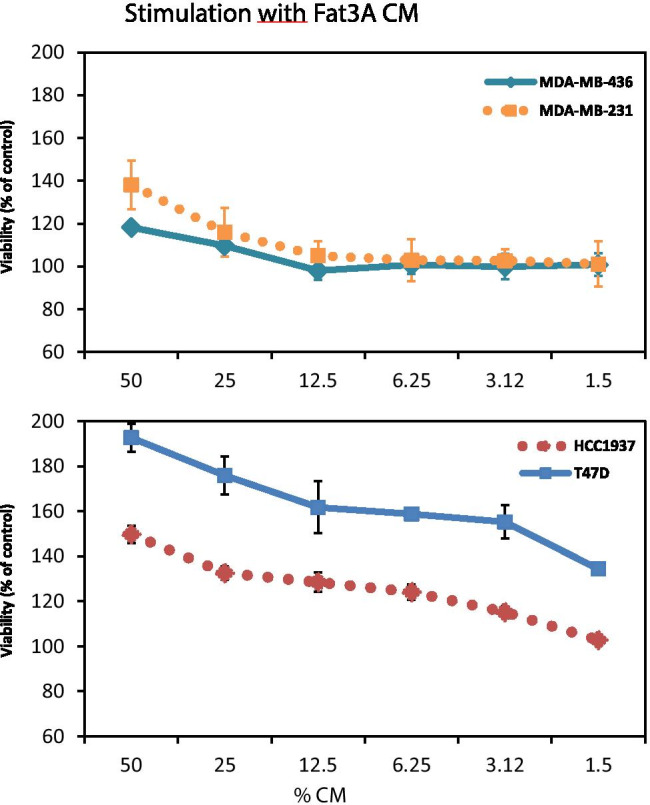
Growth stimulation of MDA-MB-231, MDA-MB-436, HCC1937 and T47D with conditioned media derived from ADSC cell line Fat3A (medium control set to 100%; data represent values ± SD; initial concentration 50% CM in normal medium; six dilution steps shown)

ADSCs were cultivated from lipoaspirates and this cell population was tested for purity by flow cytometric detection of the specific markers CD73, CD90, CD105 and absence of CD34 as described previously [7]. CM of the ADSC cultures were applied to proliferation assays in twofold dilutions to the respective breast cancer cell lines (Fig. 1).

**Fig. 2:**
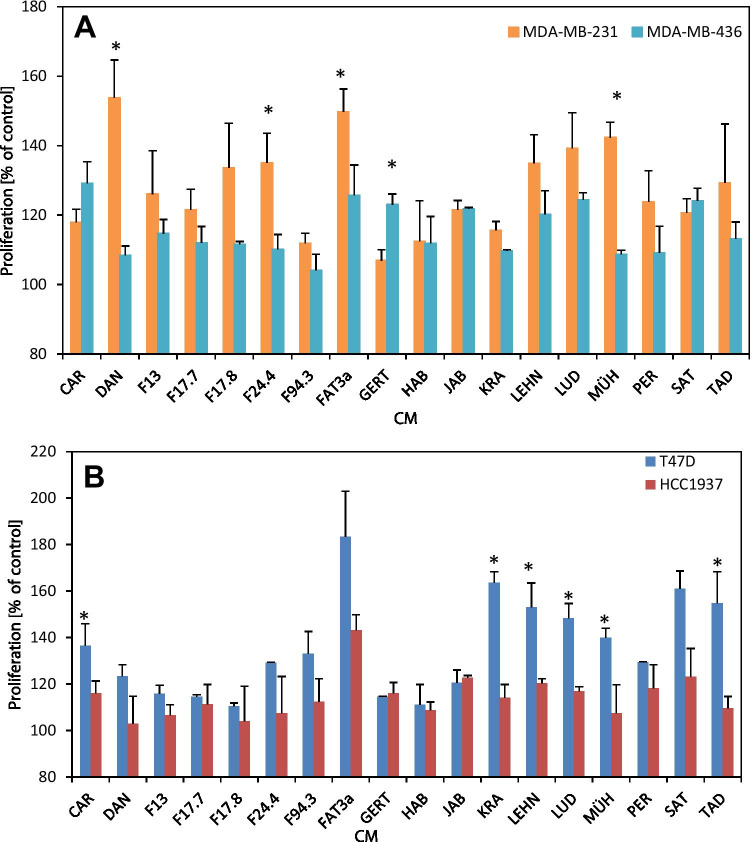
Stimulatory effect of CM derived from a panel of ADSC cell lines on MDA-MB-231 and MDA-MB-436 (**A**) and on T47D and HCC1937 (**B**) BC cells. Statistical significance is indicated by an asterisk. Data are shown as mean values ± SD. The highest stimulatory effects are shown observed for the 50% initial concentration of CM in regular medium

The results show that the CM of ADSCs result in a dose-dependent stimulation of the proliferation of the cancer cell lines and that, in case of the Fat3A CM, this effect is more pronounced for the nonmutated cell lines MDA-MB-231 and T47D versus the BRCA1/2-mutated cell lines MDA-MB-436 and HCC1937, respectively (Fig. 1). The proliferation tests for the 4 breast cancer cell lines were extended to CM derived from ADSCs of 18 different patients (Fig. 2A and 2B). Figure 2A. shows a summary of the growth stimulation experiments for control MDA-MB-231 and BRCA-mutated MDA-MB-436 employing ADSC-CM of 18 preparations. Comparison of the effects found for MDA-MB-231 and MDA-MB-436 revealed that only 1/18 samples (GERT) gave a significant higher response for the BRCA-mutated cell line whereas 4/18 CM gave an opposite signal (Fig. 2A). The T47D BC control cell line was grouped with the mutated HCC1937 line according to their similar growth morphology. In this case, the ADSC-CM revealed a higher growth stimulation for the nonmutated T47D line that was statistically significant for 6/18 ADSC cultures (Fig. 2B).

### Effects of ADSC-conditioned Media on Cancer Cell Migration

**Fig. 3:**
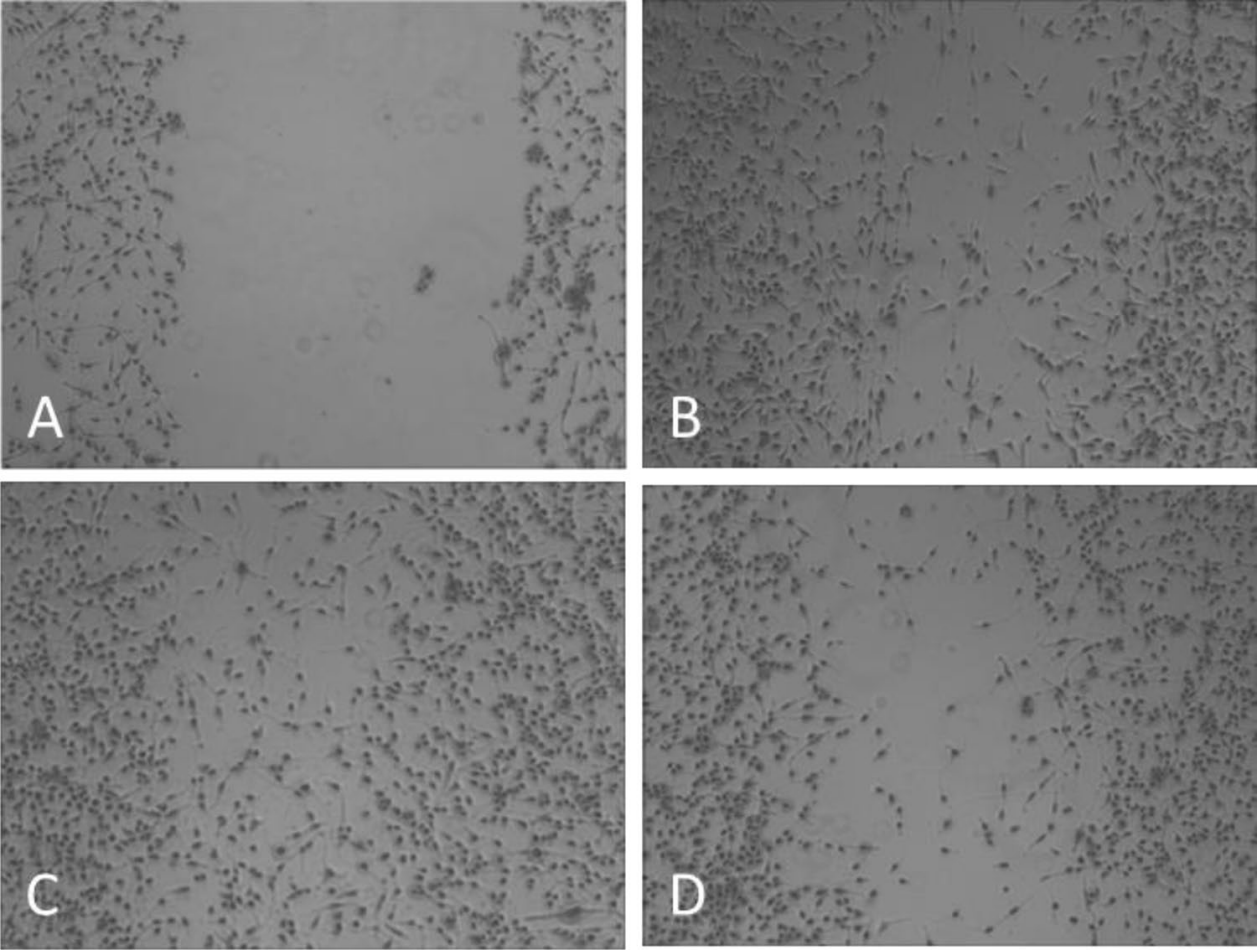
Light microscopy picture of the migration assay of MDA-MB-436. **A**) medium control showing the scratch area; **B**) medium control at day 2; **C**) effect of F13 CM at day 2;** D**) effect of HAB CM at day 2

The migration of the cancer cell lines was investigated in migration assays supplemented with control medium and ADSC-CM, as described previously [7]. T47D cells exhibited no significant migration within 2 days and were omitted. Confluent monolayers of the cells were scratched and the so-called wound healing process monitored microscopically for 2 days (Fig. 3). Within 2 days, cells migrate into the empty tissue culture flask area and this movement was retarded upon supplementation of the cultures with F13 and HAB CM.

**Fig. 4:**
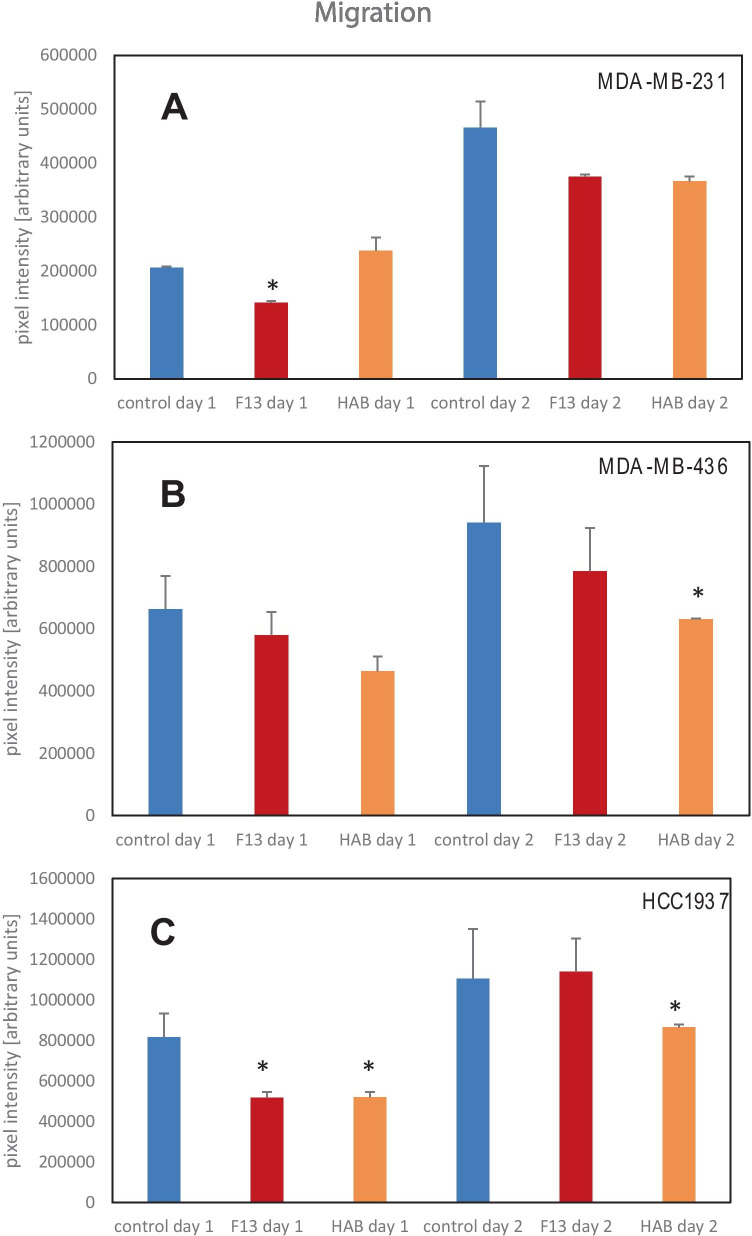
Analysis of the migration assays of MDA-MB-231 (**A**), MDA-MB-436 (**B**) and HCC1937 (**C**). The cell lines were stimulated with CM derived from ADSC F13 and HAB. Statistical significance is indicated by *. Data is shown as mean values ± SD and represent the actual area newly covered by migrating cells

A quantitative determination of the migratory capacity was carried out using image analysis. For MDA-MB-231, addition of F13 and HAB media showed a trend of lower migration which was significant for F13 and day 1 (Fig. 4A). For the BRCA-mutated MBA-MD-436 a similar retarding effect was detectable which was significant for HAB and day 2 (Fig. 4B). Migration of BRCA-mutated HCC1937 cells was retarded in a similar manner with statistical significance for both supernatants at day 1 and HAB media for days 2 (Fig. 4C).

**Fig. 5:**
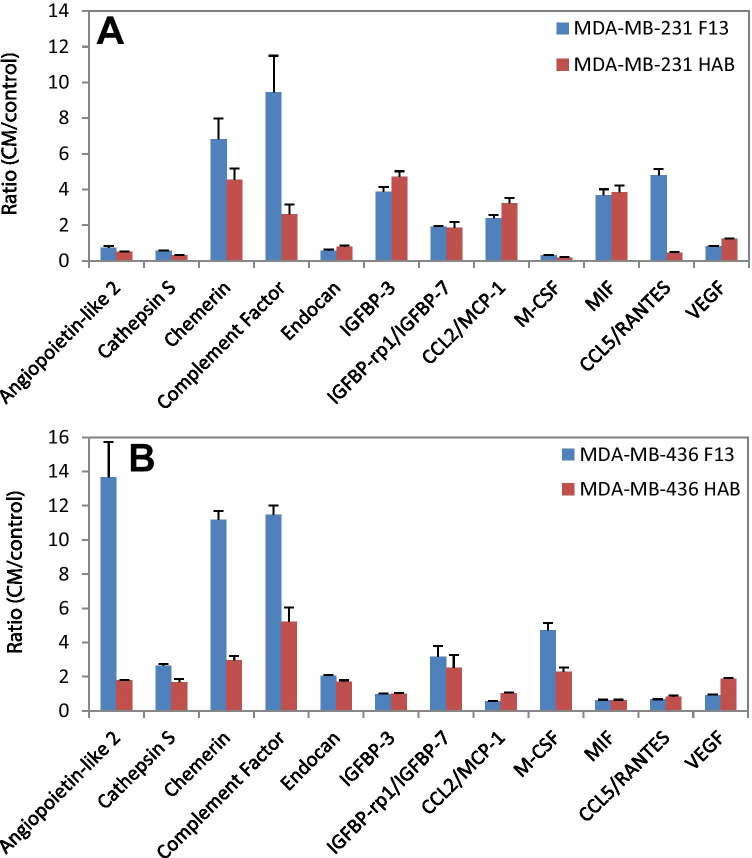
**A** and **B** Ratio CM/medium of protein expressions of MDA-MB-231 and MDA-MB-436 pretreated with CM of F13 and HAB ADSCs, respectively (mean values ± SEM). Significantly different adipokines are shown

**Fig. 6:**
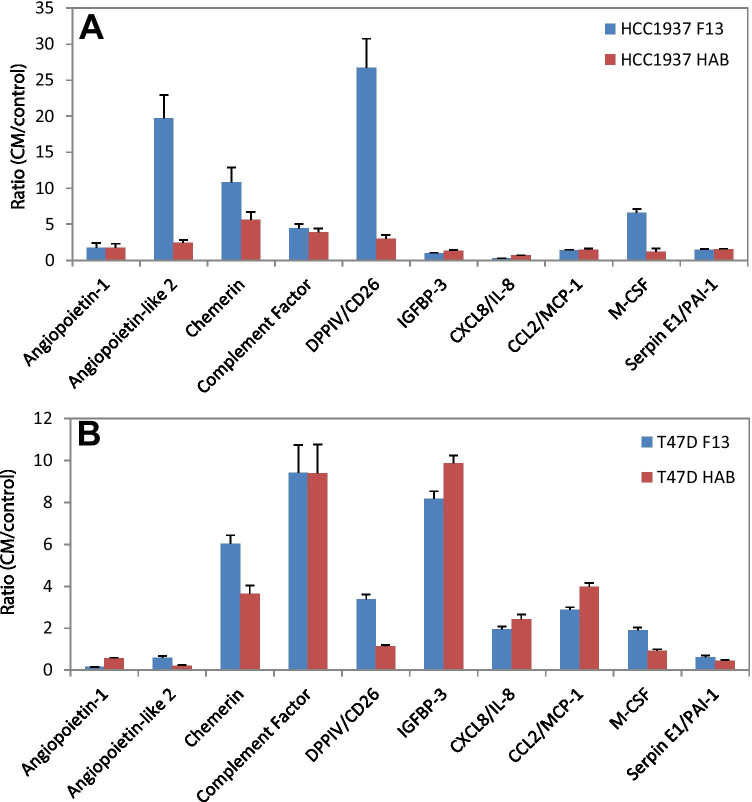
**A** and **B** Ratio CM/medium of protein expressions of HCC1937 and T47D pretreated with CM of F13 and HAB ADSCs, respectively (mean values ± SEM). Significantly different adipokines are shown

### Analysis of Adipokine-related Proteins Changed by ADSC stimulation

The four breast cancer cell lines were preincubated with 50% of the respective ADSC-CM and after further incubation analyzed for the expression of proteins using an adipokine proteome profiler array. Of the 58 adipokines analyzed, 12 proteins are shown for the MDA-MB-231/MDA-MB-436 pair and 10 proteins for the HCC1937/T47D pair, respectively (Fig. 5 and Fig. 6). The relevant differences under investigation were the diverse responses of wildtype and BRCA-mutated BC cell lines to CM of the ADSCs. Angiopoietin-like 2 showed higher induction in response to F13 CM in MDA-MB-436 cells whereas chemerin and complement factor yielded a similar response in both cell lines for both CM used. Elevations in IGFBP-3, CCL2, MIF and CCL5 were more pronounced in MDA-MB-231 versus MDA-MB-436 in contrast to the expression of M-CSF. Induction of angiopoietin-like-2, chemerin, complement factor and DPPIV was higher in HCC1937 versus T47D in contrast to IGFBP-3. With the exception of angiopoietin-like-2 and DPPIV in the BRCA-mutated breast cancer lines, the other differences in ADSC-induced proteins are of minor quantitative nature.

## Discussion

Breast reconstruction is a favorable option for women with a BRCA1/2 mutation who undergo risk-reducing mastectomy [12]. BRCA1 carriers have earlier-onset disease, particularly under age 50 and are more likely to develop aggressive triple-negative breast cancer than BRCA2 carriers or those who are BRCA mutation negative [13]. Bilateral prophylactic mastectomy decreases the incidence of breast cancer by 90% or more in patients with BRCA mutation. Regarding the surgical technique, nipple-sparing mastectomy is the current standard procedure that is able to optimize the oncological and aesthetic results [14]. In the absence of contraindications, all patients should undergo breast reconstruction in order to minimize the negative impact of the mastectomy. The breast reconstruction should be immediate and performed at the same time of the prophylactic mastectomy with permanent prosthesis or autologous tissues. There may be the need to resort to further aesthetic/plastic procedures after the prophylactic mastectomy to correct some imperfections or repair complications.

Among other techniques, lipofilling can be used for breast reconstruction of these patients. However, so far there are not sufficient data available to establish the safety of such a procedure in respect to tumorigenesis and tumor recurrence [5, 15]. In fact, adipocytes, preadipocytes and progenitor cells can stimulate angiogenesis and cancer cell growth. The effects of lipofilling were studied by Aroldi et al. in patients with BRCA mutations and cancer and the authors reported 3 cancer related events: two local relapses and one systemic recurrence [15]. The median follow-up from primary surgery was quite long: 60 months (range 20–93) whereas time from lipofilling was 27 months (range 10–64 months) [15]. The median number of lipofilling was 3 (range 1–6). Half of the patients had a BRCA1 mutation, 42% a BRCA2 and 8% a variant of uncertain significance in BRCA2. In another study, 18 BRCA carriers with no history of breast cancer who had undergone bilateral prophylactic mastectomy followed by breast reconstruction with lipofilling were observed [5]. A total of 36 lipofilling procedures were performed following an implant or flap, or as an exclusive fat grafting for breast reconstruction. The average number of lipofilling sessions was 1.4 with a mean volume of 108.8 ml per breast. Median follow-up was 33.0 months after mastectomy and 24.5 months after the last lipofilling intervention and no patients were diagnosed with BC during follow-up. This study with limited follow-up provides a first hint to the safety of this procedure in patients without cancer.

For the present study we employed two BRCA1-mutated cell lines, namely MDA-MB-436 and HCC1937, which lack expression of this protein, and compared these lines with matching wild-type BC cell lines MDA-MB-231 and T47D. Tests were performed to check for differences in proliferation, migration and stimulated expression of several adipokines after exposure to a number of ADSC-derived CM. Unfortunately, only a few BRCA-mutated breast cancer cell lines are available [16]. Both MDA-MB lines present as small loosely attached cells and HCC1937 and T47D represent firmly attached cells that require prolonged trypsin treatment for harvesting. The breast cancer cell lines were stimulated with CM of a large panel of ADSCs and the BRCA1-mutated cell lines revealed no higher proliferation compared to the matching wild-type breast cancer cells. The migratory capacity of the BRCA1-mutated cell lines was not stimulated by ADSC-CM, instead, a retardation was found for the HAB ADSC-CM. Since triple-negative BC lines are known to exhibit higher mobility only MBA-MD231, MDA-MB-436 and HCC1937 were compared for the effects of CM of the ADSCs on migration.

A range of adipokine-related markers were analyzed in the wildtype and BRCA1-mutated breast cancer cell lines exposed to ADSC-CM. In all cell lines, chemerin and complement factor/adipsin were induced in response to the ADSC-CM. Chemerin is a multifunctional adipokine with established roles in inflammation, adipogenesis and glucose homeostasis [17]. Chemerin is expressed in many tissues and is able to induce angiogenesis in endothelial cells. It is suggested that chemerin is important for early immune responses to infection, injury and inflammation [18]. However, the role of chemerin in cancer is not fully understood. El-Sagheer et al. detected a higher protein expression in cancerous tissue compared to healthy ones which was found to be also associated with poor survival rates. Pachynski et al. found a reduced expression of chemerin RNA in malignant breast cancer tissue compared to normal samples. Due to its ability to recruit immune effector cells based on increased gene expression chemerin may exhibits anti-cancer effects [18–20]. In breast cancer, with regard to tumor expression of chemerin receptors, this adipokine is suggested to exert a tumor-suppressive role via binding to chemokine-like receptor 1 (CMKLR1) and G protein-coupled receptor 1 (GPR1) which effect growth-inhibitory downstream signaling [17]. Complement factor D (also called adipsin) is one of the most prominent proteins in adipose cells and catalyzes the limiting step of the alternative pathway of complement activation [21]. Adipsin was demonstrated to enhance proliferation of human breast cancer patient-derived xenograft (PDX) cells and may be partially responsible for the increased proliferation triggered by ADSC-CM [22].

Angiopoietin-like 2 is prominently overexpressed in MDA-MB-436 in response to F13-ADSC-supernatant compared to MDA-MB-231 wildtype cells and the same effect is detectable in HCC1937 breast cancer cells. Angiopoietin-like 2 is a secretory glycoprotein related to angiopoietins which is expressed by many tissues and is associated with angiogenesis and inflammation [23]. The expression of angiopoietin-like 2 is elevated in obesity and related pathological conditions [24]. Both in vitro and in vivo experiments showed that the levels of angiopoietin-like 2 secreted from breast cancer cells increased with cell proliferation and cancer progression [25]. Angiopoietin-like 2 may also contribute to vasculogenesis and an important physiological property of this factor is that it increases survival and expansion of progenitor cells [26]. Of the other proteins determined, angiopoietin-1 maintains mostly the homeostasis of blood vessels which are in a quiescent state, while angiopoietin-2 plays a crucial role in malignant diseases [27]. Intracellular endocan is also an important regulator of cell growth and can also facilitate tumor growth [28]. The expression of CCL2 and CCL5 is associated with inflammation and with advanced breast cancer and tumor progression [29, 30]. MIF can promote tumor microenvironment via macrophages [31].

In HCC1937 cells exposed to ADSC-CM, DPPIV/CD26 is upregulated and IGFBP-3 shows a reduced expression. DPPIV regulates the activity of biopeptides by proteolytically cleaving a number of peptides, cytokines, and chemokines. DPPIV plays a significant role in cancer biology and inhibition of DPPIV promotes cancer metastasis via induction of the CXCL12/CXCR4/mTOR/EMT axis [32], implying a dissemination-suppressive role in cancer. The family of insulin growth factor binding proteins (IGFBPs) plays an important role as modulators of the signaling associated with insulin growth factors (IGFs) [33]. In tumors, high IGFBP-3 levels in breast cancer tissue are correlated with rapid growth and poor prognosis [34, 35]. Therefore, high expression of DPPIV and reduced expression of IGFBP-3 point to reduced metastasis and proliferation of the breast cancer cells.

BRCA1 gene has been extensively studied and more than 1600 mutations have been described with the majority of them constituting frameshifts mutations resulting in the deletion of or non-functional protein [36, 37]. One investigation studied the effect of an ADSC line in which BRCA1 was eliminated using the CRISPR/Cas9 technique on the interaction with wildtype MDA-MB-231 breast cancer cells [11]. This BRCA1-negative ADSCs were reported to induce a more aggressive phenotype in MDA-MB-231, that is already recognized as highly invasive breast cancer cell line. BRCA1 has a tumor suppressor function and it has been documented previously that knocking down the expression of BRCA1 in BRCA1 wild-type cells resulted in an increase in the rate of proliferation, increase in the propensity to grow in soft agar, to migrate and to invade matrigel [38]. Bendera et al. studied the effect of CM of BRCA1 and BRCA- mutated or wild-type ADSC on different wildtype breast lines. The CM of the ADSCs induced the proliferation of luminal, Her2 and basal-like tumor breast lines. This proliferative effect of differentiated ADSCs and their estrogenic signaling was independent of the BRCA mutation status. Furthermore, the tumor stroma of BRCA1/2 mutated patients was found to be severely altered and to promote tumor growth and dissemination [39]. It was concluded that cells in lipofilling should only be used after removal of cancer [40].

The present investigation studied the effects of wildtype ADSCs on wildtype and BRCA1-mutated breast cancer cell lines and found no major difference of the effects of CM derived from a panel of ADSCs. For the case of possible adverse effects of BRCA1-mutated ADSCs, allogeneic ADSCs may be used which are known to exhibit low immunogenicity [41]. In fact, ADSCs beyond passage 1 failed to elicit a T lymphocyte response and late passage ADSCs actually suppressed the mixed lymphocyte reaction, thus supporting the feasibility of allogeneic human ADSC transplantation [42]. The own BRCA1/2 mutated ADSCs may be removed by their adherence to plastic surfaces and replaced by allogeneic cells during lipofilling. Prophylactic mastectomies and, as a result, breast reconstruction is also performed more frequently and making lipofilling more often eligible to improve the aesthetic results. Although guidelines disapprove fat grafting in patients with positive familial history or genetic alteration in BRCA1/2 genes, increasingly clinical and experimental evidence support lipofilling for these patients [10, 42].

## Conclusion

BRCA1/2 mutations result in an increased incidence of breast and other cancers. Frequently, carriers of these mutations chose prophylactic mastectomy but material from liposuctions is not routinely used for reconstructive surgery for these patients due to possible increased tumor initiation or recurrence. The present study compared the interaction of adipose-derived stromal cells (ADSCs) on wildtype and BRCA1-mutated breast cancer cell lines and found no increased protumor effects of CM of ADSCs.

## Data Availability

Available on reasonable request.
